# Impact of increased nest temperature on incubation behavior and female health in Eurasian Blue Tit

**DOI:** 10.1093/beheco/arag016

**Published:** 2026-02-21

**Authors:** M García-del Río, S Merino, F Castaño-Vázquez, Y Merino, M Fuertes-Recuero, A Cantarero

**Affiliations:** Department of Evolutionary Ecology, National Museum of Natural Sciences, CSIC, Calle de José Gutiérrez Abascal 2, Madrid 28006, Spain; Department of Evolutionary Ecology, National Museum of Natural Sciences, CSIC, Calle de José Gutiérrez Abascal 2, Madrid 28006, Spain; Department of Evolutionary Ecology, National Museum of Natural Sciences, CSIC, Calle de José Gutiérrez Abascal 2, Madrid 28006, Spain; Department of Evolutionary Ecology, National Museum of Natural Sciences, CSIC, Calle de José Gutiérrez Abascal 2, Madrid 28006, Spain; Department of Physiology, Veterinary School, Complutense University of Madrid, Avenida Puerta de Hierro s/n, Madrid 28040, Spain; Department of Physiology, Veterinary School, Complutense University of Madrid, Avenida Puerta de Hierro s/n, Madrid 28040, Spain

**Keywords:** blood parasites, climate change, *Haemoproteus*, *Lankesterella*, parental care, temperature, tradeoffs

## Abstract

The increase in temperature associated with climate change may influence reproductive costs in birds, particularly during the incubation period, which is temperature-dependent and crucial for embryo development. Female birds are usually solely responsible for incubation and should face a tradeoff between the time spent incubating and foraging or performing other self-maintenance activities outside the nest. Here we studied the effects of temperature on incubation behavior, female health and offspring fitness-related traits of Blue Tits (*Cyanistes caeruleus*). To that end, we experimentally increased the temperature inside nest boxes used by Blue Tits by an average of 3.29 °C as compared to control nests. Females in heated nests spent less time incubating with more frequent incubation sessions as compared to females from control nests. Furthermore, fewer females attending heated nests were infected at 3 d of nestling age, and those infected had lower intensity of blood parasite infections and were infected by a lower number of different parasites. In addition, females from control nests with longer incubation periods exhibited higher infection intensities of *Haemoproteus* and *Lankesterella.* This could be due to improved immune resource allocation as incubation effort decreased. However, 10 d later, most of the effects on blood parasites disappear, and we found no effect of treatment on females' body mass or breeding success, suggesting that control females achieve similar reproductive success by investing more time incubating, at the cost of their health. These results indicate that variations in nest temperature can have significant short-term effects that might have consequences for fitness.

## Teaser

Rising temperatures from climate change may reduce the time female birds spend incubating eggs, allowing them more time for self-care. An experimental study on Blue Tits (*Cyanistes caeruleus*) found that warmer nests led to less incubation effort and lower parasite levels in females. However, these benefits appear to be temporary and reproductive success remained unchanged. These findings show that nest temperature changes during the incubation period can impact bird behavior and health, with short-term fitness effects.

## Introduction

Bird reproduction involves several energy-intensive activities, such as nest building, egg production and incubation, and offspring brooding and provisioning. These are all critical stages in the avian life cycle, during which birds invest substantial energy to maximize their reproductive success (Bell 1980; Barreda et al. 2026). Among these activities, avian incubation is highly sensitive to temperature, as developing embryos require a narrow range of optimal temperatures, typically between 36 and 38 °C, to develop properly (Drent 1975; Webb 1987). Therefore, ambient temperature is crucial as it strongly impacts energy expenditure during incubation. Under colder conditions, females are expected to spend more time at the nest to maintain suitable temperatures for the developing embryos, while warmer environments may reduce these energy demands (Conway and Martin 2000).

Climate change models predict a warmer climate and greater variability in weather components, such as temperature and rainfall, across the globe in the coming decades. Furthermore, recent years have seen a marked increase in the frequency of heatwaves, which are typically characterized by abrupt and substantial temperature rises (Robinson et al. 2021). As global temperatures rise, these temperature-dependent effects on incubation may become even more significant. In fact, warmer temperatures are predicted to alter not only the incubation period but also other aspects of reproductive success, including the timing of breeding, clutch size and nestling development (Both and Visser 2001, 2005; Vaugoyeau et al. 2017). For example, many bird species have advanced their laying date in response to the increase in spring temperatures to try to adapt to the change in peak food availability, thus affecting nestling survival (Visser et al. 2006; Merino et al. 2025). However, our understanding of the ecological responses to climate change in different organisms is still evolving (Dunn et al. 2010; du Plessis et al. 2012; Halupka and Halupka 2017). To predict how populations and species will adapt to a rapidly changing world, it is crucial to gain insight into how animals respond to changing environments. Birds, in particular, provide an excellent model for studying the impacts of climate change on life-history traits. Although many bird species build well-insulated nests, eggs are often exposed to external ambient temperatures outside this optimal range, especially when the parent birds are absent. In such cases, parents must carefully regulate the thermal environment of the nest to prevent overheating or cooling of the eggs (Carey 2002; Deeming 2002). Incubating parents often must balance their own energy needs with the thermal needs of developing embryos (Bambini et al. 2019; Bulla et al. 2015), and these competing demands limit both daily nest attendance and the time spent foraging (Tinbergen and Williams 2002). In many cases, incubation is performed solely by the female. This can be compensated, in part, with the assistance of males, since food provisioning by males during incubation is more frequent when the conditions are worse, eg the temperature is lower (Amininasab et al. 2017).

On the other hand, studies exploring the impact of temperature manipulation during incubation have sometimes produced contradictory results. For instance, nest heating has been shown to increase, decrease, or have no effect on female attentiveness across different studies (Reid et al. 1999; Magrath et al. 2005; Londoño et al. 2008; respectively). The effect on female condition also varies, with some research reporting weight gain (Pérez et al. 2008) while others found no change (Cresswell et al. 2004) or even weight loss (Magrath et al. 2005). Meanwhile, increased nest temperatures can result in both positive and negative effects on offspring condition and survival. In some studies, heated nests produced larger, healthier nestlings (Pérez et al. 2008; Arct et al. 2022), while in others, increased temperatures during incubation reduced fledging success (Mueller et al. 2019).

Another important factor to consider during energetically demanding life-history stages, such as the incubation period, is parasitic infection, which is expected to be particularly costly as it can disrupt the host's allocation of resources (Sheldon and Verhulst 1996). There is some evidence indicating that reproductive effort may compromise or alter immune function (Norris and Evans 2000; Krams et al. 2017), which can heighten the risk of infection (Festa-Bianchet 1989; Cizauskas et al. 2015). Similarly, a decrease in female body condition due to disease can lead to increased allocation of resources towards immune defense, thereby reducing the energy available for reproduction (Moss and Camin 1970; Vandegrift et al. 2008). Numerous parasites infect birds, including haemoparasites, such as protozoa like *Trypanosoma* and those from the phylum Apicomplexa, such as *Haemoproteus*, *Leucocytozoon*, *Plasmodium* and *Lankesterella*, as well as certain multicellular organisms like filarial nematodes. In general, these parasites cause chronic infections in wild birds with relapses occurring during stressful situations for the hosts (Møller 1997; Merino et al. 2000; Scheuerlein and Ricklefs 2004; Valkiūnas 2005), further intensifying their immediate health effects and impacts on individual reproductive success (Hamilton and Zuk 1982).

Birds, being homeothermic animals, maintain a relatively constant body temperature through thermoregulatory mechanisms such as panting (De Juana 1992). Therefore, increases in nest box temperature are unlikely to affect their core body temperature directly, and consequently, the thermal environment experienced by blood parasites within the host. However, changes in the nest microclimate may still indirectly influence parasite infection in incubating females. Specifically, the energetic demands of incubation vary with clutch temperature (Amininasab et al. 2016), and the energy required for clutch thermoregulation depends on the ambient nest temperature. These energy tradeoffs can affect the female´s body condition (García-del Río et al. 2025) and/or the allocation of resources and energy to immune responses (Sheldon and Verhulst 1996), potentially increasing their susceptibility to blood parasites. This mean that changes in nest-box temperature can be expected to either benefit or impair females in terms of the energy they must invest in incubation, thereby indirectly affecting their health. Under this scenario, nest temperature first affects the female's energy balance, and any resulting changes in health or immunity subsequently influence parasite infection. Previous experimental studies have shown that a reduction in parasitaemia can lead to increased reproductive success and survival (Merino et al. 2000; Marzal et al. 2005; Martínez-de la Puente et al. 2010). Furthermore, this benefit has also been linked to an increase in the time females devote to incubation (Schoepf et al. 2022; García-del Río et al. 2026a), demonstrating that females adjust their parental investment based on their health.

Therefore, several factors influence parental investment, and these could interact in various ways, affecting individuals differently. Given this complexity, we manipulated the temperature during incubation inside nest boxes used by Blue Tits *Cyanistes caeruleus*, to analyse the potential effects on female incubation behavior and assess the consequences for the health, in terms of infection by blood parasites, and for offspring fitness-related traits.

## Methods

### Study population

This study was carried out during the 2022 bird breeding season in a montane deciduous forest of Pyrenean Oak *Quercus pyrenaica* located in Valsaín (Segovia, central Spain, 40°53′74″N, 4°01′W, 1,200 m above sea level). A Blue Tit population breeding in wooden nest boxes hanging from tree branches about 4 m above the ground has been studied in this area since 1991 (Sanz 2002; Tomás et al. 2006). Each breeding season, nest boxes are periodically inspected to determine reproductive parameters including laying date, clutch size, hatching date and brood size at fledging (Merino et al. 2000; Tomás et al. 2007).

The Eurasian Blue Tit is an insectivorous cavity-nesting bird widely distributed across the western Palearctic (Cramp and Perrins 1993) that readily uses nest boxes for breeding. Most Blue Tit populations are sedentary and, during the breeding season, females can be easily differentiated from males by the presence of a brood patch on the abdomen. Also, they present a slightly sexually dichromatic plumage, with males being more intensely colored than females (Cramp and Perrins 1998). In this population, females lay a single clutch with an average of 9.1 eggs producing 7.8 nestlings (Fargallo and Johnston 1997; Cantarero et al. 2013b).

### Experimental design

We experimentally manipulated the temperature inside nest boxes occupied by Blue Tits during the incubation period. Before experimental manipulation, nest boxes were visited daily after the seventh egg was laid to determine the beginning of incubation. Nests were grouped in pairs according to laying date and clutch size (±1 d and ±1 egg), without significant differences (Mann–Whitney test, *P* > 0.05). Each nest box was randomly assigned to an experimental category: heat treatment or control group. A total of 44 nest boxes (22 heated and 22 control) were included in the study.

On the first day of incubation, heated nests were equipped with heat mats made of plastic (70 × 70 mm, 5 V/3.5 W; thermo Flächenheizungs GmbH, Germany), for about 12 d (10 to 16 ± 1.286; from day 1 of incubation to hatching day). These heat mats provide heat by converting electrical energy into heat energy through a resistive element, which then radiates heat onto the surface of the mat. For each of these nests, a heat mat was placed on the floor of the nest box and separated from the nest material by a metal grid (García-del Río et al. 2025). Heat mats were connected through a cord to 9-V power banks that were replaced daily or every 2 d with fully charged ones to maintain heat production 24 h a day without interruption. Metal grids and cords were also installed in control nests during the experiment. All the nest boxes were also fitted with sensors, located on the rear wall of the nest box at the level matching the position of the cup of the nest where eggs are placed, that registered both temperature and relative humidity every hour during the experimental period (Thermochron DS1923; 6 × 17 mm, temperature range: −20 to 85 °C; resolution 0.0, 625 °C; humidity range: 0 to 100% with a resolution of 0.04%; Maxim IC, USA). On the hatching date, heat mats were disconnected, and sensors were removed.

On day 10 of the incubation period, in order to analyse the females' incubation behavior, we recorded nest activity for about 90 min (94.70 ± SE 9.31 min, *N* = 44) with digital video cameras (Sony Dcr-sr190, with an extra battery Sony np-fh100) placed 20 m away from the nest box tree and recording an area of approximately 1 m around the nest-box. We selected day 10 of incubation for behavioral observations to ensure that females had been exposed to the treatment for a sufficient duration and that any potential effects on behavior had time to be developed. A 90-min observation period was deemed sufficient to capture meaningful variation in incubation behavior, as supported by previous studies (eg, Diez-Méndez et al. 2021; Bueno-Enciso et al. 2017; Nord and Nilsson 2012). All films were recorded between 08:00 and 13:00 h. No nests were abandoned as a result of the manipulation procedures or filming.

On day 3 of nestling age (hatching date = day 0), females were captured in the nest box during the daytime, using a conventional nest box trap set at the entrance. A blood sample was extracted from the brachial vein, immediately smeared, and air-dried to assess the presence of parasite infections at the end of the experiment. The body mass of females was recorded with an electronic balance to the nearest 0.1 g and tarsus length was measured with a digital caliper to the nearest 0.1 mm. Additionally, nestlings from each nest were counted and weighed together as an indicator of the average mass of the brood. To assess the potential effects of the experiment once that nestlings enter the deceleration phase of growth, females were recaptured on day 13 of nestling age, and a second blood sample was taken to evaluate parasite infections. Additionally, the body mass of both females and nestlings was measured for the same purpose. These time points were selected since these days were considered the earliest and latest, respectively, at which nestlings could be handled without triggering abandonment or premature fledging. All birds were supplied with individually numbered rings for identification.

### Behavioral data analysis

Recordings were scored in the free VLC Media Player software (VideoLan 2006). To ensure consistency and avoid bias, all analyses were carried out by the same person, who was blind to the treatment assignment. We estimated the average duration in minutes of incubation sessions, as well as the percentage of time spent by the female inside the nest-box or “egg attendance”, which encompassed the time devoted to incubating and turning the eggs (Cantarero et al. 2013a). The hourly frequency of male feedings (male feedings per hour) was also determined for each recording. We found no significant differences in the timing of the filming between the two treatments (Mann–Whitney test, *U* = 238.5, *P* = 0.934).

### Blood parasite quantification

The blood smears were stained with Diff-Quik (PanReac AppliChem) in the laboratory. Searching for parasites in blood smears was conducted under an Olympus BX-41 microscope as follows. Half a smear was scanned at 200 magnifications in search of large parasites such as *Trypanosoma* and microfilariae, whereas intra-erythrocytic parasites, such as *Haemoproteus, Leucocytozoon* and *Lankesterella* were searched under oil immersion at 1,000 magnification. We were unable to obtain blood samples from some females in both captures, so we had 20 samples of females from heated nests and 21 of control females at day 3 of nestling age, and 15 samples of females from heated nests and 16 of control females at day 13 of nestling age. Due to the limited occurrence of *Trypanosoma*, *Leucocytozoon* and microfilaria haemoparasites, quantifying infection intensities proves challenging. Consequently, we opted for a presence-absence index of parasites and the number of different parasites, when conducting analyses of all blood parasites. In the case of *Haemoproteus* and *Lankesterella* haemoparasites, we were able to calculate the intensity of infection as the number of parasites per 2,000 erythrocytes, counting the number of erythrocytes per field (Godfray et al. 1987). We first examined 50 fields to search for the parasites, and in the case that they were detected after that, the quantification was performed. If no parasites were detected in 2,000 erythrocytes, the quantification was extended to 10,000. Lastly, in cases in which the parasite was detected in the 50 fields but not found when counting 10,000 erythrocytes, it was assigned a value of 0.1 parasites per 2,000 erythrocytes (Fargallo and Merino 1999). Although molecular methods are more sensitive for detecting low-level infections, microscopy was selected as the sole diagnostic approach since it is a robust method for assessing reductions in infection due to experimental treatment (Merino et al. 2000), consistent with the aim of the present study.

### Statistical analyses

To test the effect of the experimental manipulation on nest box temperature, we averaged the values recorded per nest box and then calculated the average value for the experimental period (from day 1 of incubation to hatching date). Thus, we obtained a continuous variable with an average temperature per nest box for the experimental period. After checking if the variables complied with normality assumptions, ANOVA was used to test if the experimental treatment influenced temperature.

To investigate behavioral changes in incubation due to experimental manipulation, we used the following variables: “Percentage of incubation time” as variable of time spent incubating compared with the total time recorded (%), “Average duration of incubation sessions” as variable of the total minutes a female spent inside the nest box divided by the number of times the female entered the nest box (in minutes), and “Incubation sessions per hour” and “Male feedings per hour” as variables indicating the number of incubation sessions and male feedings per hour respectively. Here, we used generalized linear models (GLMs) with each of the previous variables mentioned as dependent variables, the experiment as a fixed factor and the “experimental pair” as a random factor. Because the dependent variables did not comply with normality assumptions, we could transform “Average duration of incubation sessions” using the square root. This model had a normal distribution and therefore employed identity link function. For the rest of the variables that could not be transformed (“Percentage of incubation time”, “Incubation sessions per hour” and “Male feedings per hour”), we used models with Gamma distribution and log link function. This distribution was selected because the variables are continuous, representing proportions (decimal values between 0 and 1) or means (number of sessions in each hour) and exhibited variances greater than their means, indicating a right-skewed distribution.

To test for the effect of the experimental manipulation on (i) female's body mass, (ii) average nestling body mass on day 3 of nestling age, (iii) hatching success (calculated as the proportion of eggs that successfully hatched) and (iv) fledging success (computed as the percentage of hatched nestlings that successfully fledged), we used GLMs with these variables as dependent variables and the experiment as a fixed factor, and the tarsus length as covariate in the (1) female's body mass model. The (i) female's body mass and, (ii) brood body mass on day 3 of nestling age models, complied with normality assumptions and we applied an identity link. Brood body mass on day 3 of nestling age was previously transformed using the Napierian logarithm (Ln) to comply with normality assumptions. For the variables (iii) hatching success and (iv) fledging success, we used models with Gamma distribution and log link function.

To test for the potential effects of the experiment on female's and brood's body mass variation between days 3 and 13 of nestling age, once that nestlings enter the deceleration phase of growth, we used generalized linear mixed models (GLMMs) with the female's and brood's body mass of both dates as dependent variables (all with normal distribution and assuming identity link function), and the experiment, the date (first and second capture) and their interaction as fixed factors, and the tarsus length as covariate in the female's model. The interaction is included to explore whether there were differential effects of breeding date between groups. We structured the data as repeated measures, specifying that each nest had two measurements.

Lastly, we investigated the effect of the experiment on female's blood parasite infections on day 3 of nestling age. First, we tested if there was an effect on the presence or absence of blood parasite infections studied (altogether) by using a Pearson's Chi-square test with 10,000 Monte Carlo permutations. Then, we repeated this same analysis, but this time on the number of different blood parasites detected (from 0 to 3), to investigate the potential effect on mixed infections. It was not possible to test the difference between days 3 and 13 using a GLMM due to the distributional properties of the variables, which did not meet the model assumptions. We did these same analyses with data obtained at the second capture, the day 13 of nestling age. In addition, we tested for the effect of the experiment on the infection intensity of *Haemoproteus* and *Lankesterella*, using their intensities on day 3 of nestling age as dependent variables and the experiment as a fixed factor, in two different GLMs, with a negative binomial distribution and log link function. This distribution is appropriate for the analysis of the aggregated distribution of parasites. In addition, we applied the same Generalized Linear Model (GLM) using *Haemoproteus* and *Lankesterella* infection intensities on day 3 of nestling age but including the “average duration of incubation sessions (min)” and its interaction with the experimental treatment added as a fixed factor, to test for the direct influence of behavior on infection intensity.

Finally, to test for potential effects of the experiment on variation in the intensity of blood parasites infection in females between days 3 and 13 of nestling age, once that nestlings enter the deceleration phase of growth, we used GLMMs with the infection intensity data for *Haemoproteus* and *Lankesterella* from the two sampling dates as dependent variables (with a negative binomial distribution and log link function), and the interaction between experiment and the date (day 3 and day 13 of nestling age) as fixed factor. We did not include the main effects of the factors because they had already been tested in the GLMs. We structured the data as repeated measures, specifying that each nest had two measurements. On day 3 of nestling age, we were able to collect a total of 42 blood samples from the females (21 experimental pairs). As some females could not be recaptured or a blood sample could not be obtained on day 13 of nestling age, the sample was reduced by 5 experimental pairs, that is, a reduction of 10 females (*N* = 32), resulting in a total of 64 samples across both days, considering only the females with samples at both dates. Thus, repeated measures analyses included different sample sizes than analyses of variables at day 3 of nestling age. Graphics and statistical analyses were performed in SPSS (released 2020, IBM SPSS Statistics for Windows, Version 27.0.1.0: IBM Corp., Armonk, NY, USA) and R version 4.5.1 (R Core Team 2024).

## Results

### Climatic variables

**Figure 1:**
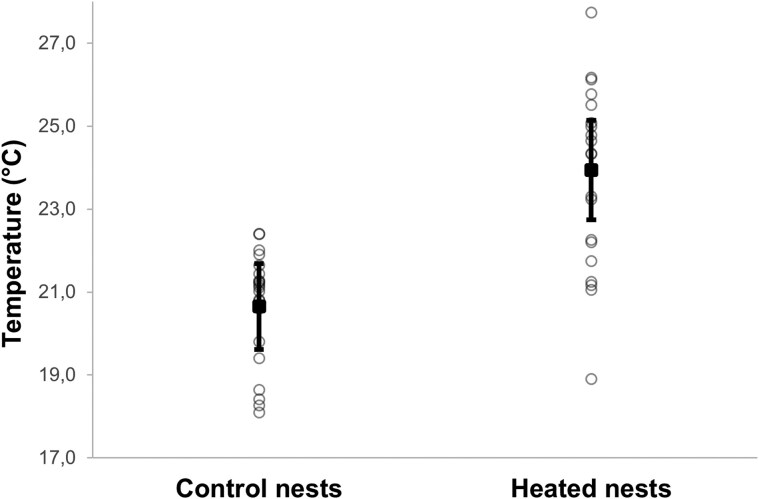
Differences in temperature inside Blue Tit nest boxes assigned to different treatments. Estimated marginal means ± intervals of confidence at 95% are shown, with raw data shown as individual points.

The mean temperature in the heated nests (23.94 °C ± 0.49 SE) was significantly higher than in the control nests (20.65 °C ± 0.28 SE), specifically 3.29 °C (±0.38 SE) higher on average (ANOVA, *F*_1,42_ = 33.681, *P* < 0.001, Fig. 1).

### Incubation behavior

**Figure 2:**
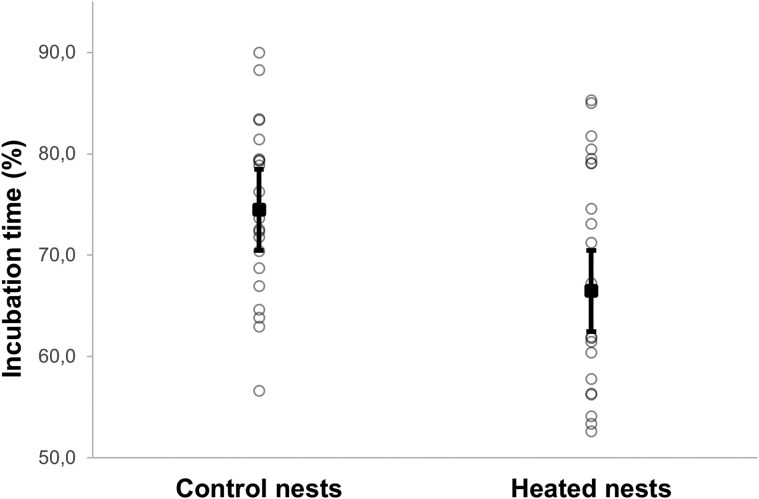
Percentage of incubation time by female Blue Tits from nest boxes assigned to different treatments. Estimated marginal means ± intervals of confidence at 95% are shown, with raw data shown as individual points.

The proportion of incubation time (%) and the average duration of incubation sessions (min) were significantly greater in control than in heated nests (Table 1; Fig. 2). The number of incubation sessions was significantly greater in heated than in control nests (Table 1). However, there were no significant effects of the experiment on the number of male feedings per hour (Table 1; Figure S1).

**Table 1:** Differences between treatment groups in incubation variables

	Control	Heat	Statistic	*P*
**Percentage of incubation time (%)**	74.48 ± 2.66	66.47 ± 2.37	*F*_1,42_ = 5.088	**0**.**029**
**Average duration of incubation sessions (min)**	14.06 ± 1.01	10.89 ± 1.01	*F*_1,42_ = 7.394	**0**.**009**
**Incubation sessions per hour**	3.48 ± 0.234	4.44 ± 0.298	*F*_1,42_ = 6.912	**0**.**012**
**Male feedings per hour**	2.55 ± 0.332	2.05 ± 0.267	*F*_1,42_ = 1.336	0.254

Estimated marginal means ± SE are shown. Statistics correspond to the results of GLMMs. Significant results are marked in bold.

### Body condition and reproductive success

The experimental manipulation of temperature during the incubation did not have an overall effect on reproductive parameters. Hatching success (GLM, *F*_1,42_ = 0.115, *P* = 0.736; Figure S2), fledging success (GLM, *F*_1,42_ = 0.023, *P* = 0.881; Figure S3), nestling body mass at day 3 (GLM, *F*_1,42_ = 1.450, *P* = 0.235) and female's body mass (GLM, *F*_1,42_ = 0.928, *P* = 0.341) at day 3 were unaffected by the experiment (see Table S1 for more information on results of these GLMs).

Considering the data from days 3 and 13 of nestling age, we also found no effect of the treatment and the interaction between the treatment and the date on the body mass of females (GLMM, *F*_1,84_ = 0.684, *P* = 0.410; and *F*_1,84_ = 0.485, *P* = 0.488; respectively; Figure S4) and nestlings (GLMM *F*_1,84_ = 0.733, *P* = 0.394; and *F*_1,84_ = 0.014, *P* = 0.907; respectively; Figure S5). However, we did find an effect of the date on both variables, with body mass increasing in nestlings from day 3 (3.035 ± 0.124 g) to day 13 (10.062 g ± 0.124 g; GLMM, *F*_1,84_ = 1,605.352, *P* < 0.001) and decreasing in females from day 3 (10.3 ± 0.082 g) to day 13 (9.8 ± 0.075 g; GLMM, *F*_1,84_ = 18.483, *P* < 0.001) as expected. See Tables S2-S5 for more information on the results of GLMs of nestling and female body mass.

### Blood parasites

The number of infected females by any parasite detected was significantly lower in those attending heated nests than in those attending control nests, both at day 3 (*χ*^2^ = 7.38; *P* = 0.0072) and day 13 of nestling age (*χ*^2^ = 4.898; *P* = 0.0444). In both tests, standardized residuals indicated that the observed frequencies in control nests and heated nests deviated significantly from those expected under independence. Specifically, noninfected individuals were under-represented in control nests and over-represented in heated nests, while the opposite pattern was observed among infected individuals. In addition, in control nests, there were significantly more females with more than one blood parasite (mixed infection) than in heated nests on day 3 of nestling age (*χ*^2^ = 12.75; *P* = 0.0033; Table 2). Standardized residuals showed that parasite count 0 was significantly under-represented in control nests and over-represented in heated nests, whereas parasite count 2 was over-represented in control nests and under-represented in heated nests. Other categories showed smaller deviations. However, on day 13 of nestling age, there were no differences between females from heated or control nests in the number of blood parasites detected (*χ*^2^ = 7.4678; *P* = 0.1042).

**Table 2:** Number of female blue tits uninfected and infected by different blood parasites on day 3 and 13 of nestling age (standardized residuals), depending on the treatment.

Date	Treatment	Number of different blood parasites			
		0	1	2	3
**3**	Heat	6 (2.72)	8 (0.12)	4 (−2.72)	2 (1.49)
	Control	0 (−2.72)	8 (−0.12)	14 (2.72)	0 (−1.49)
**13**	Heat	4 (2.21)	4 (0.11)	5 (−0.94)	2 (−1.39)
	Control	0 (2.21)	4 (−0.11)	9 (0.94)	4 (1.39)

**Figure 3:**
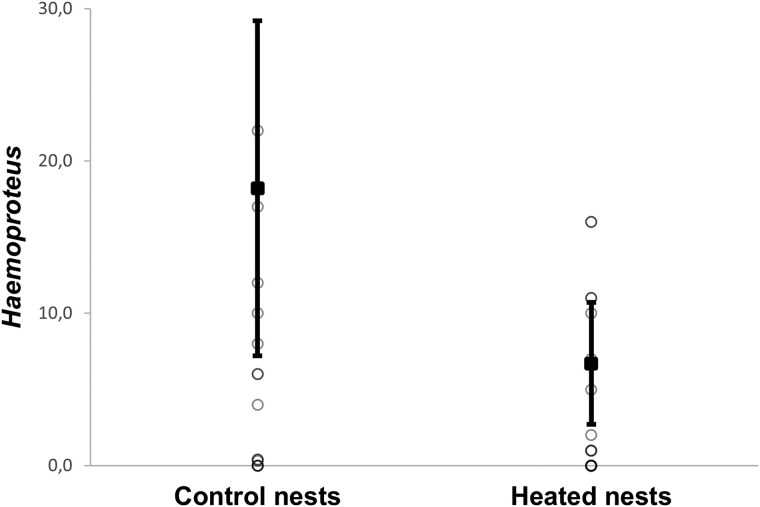
Intensity of infection by *Haemoproteus* in female Blue Tits at day 3 of nestling age by treatment. Estimated marginal means ± intervals of confidence at 95% are shown, with raw data shown as individual points.

**Figure 4:**
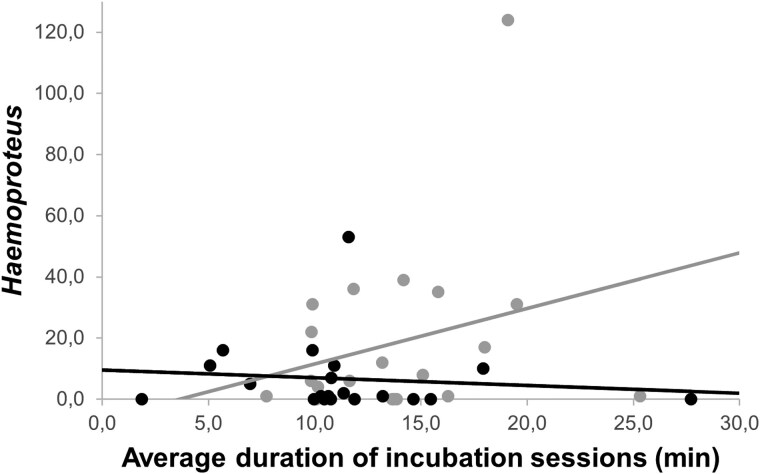
Relationship between the abundance of *Haemoproteus* in females (on day 3 of nestling age) and their average incubation session duration (min) for each treatment group. Control nests: gray color, linear *R*² = 0.073. Heated nests: black color, linear *R*² = 0.012.

**Figure 5:**
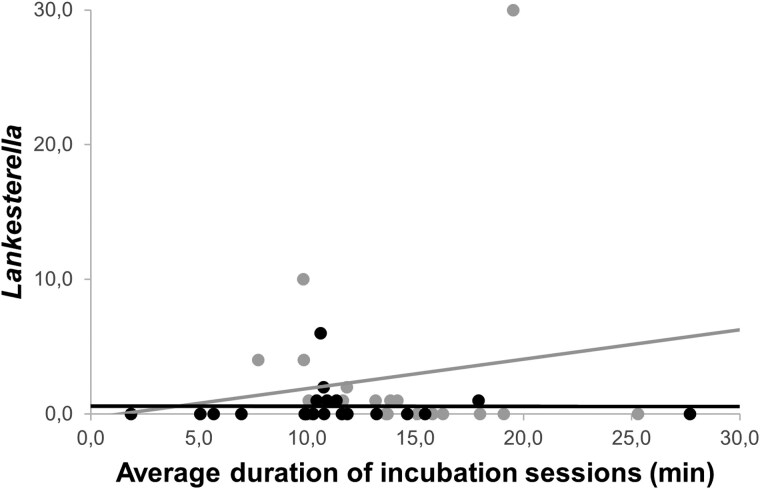
Relationship between the abundance of *Lankesterella* in females (on day 3 of nestling age) and their average incubation session duration (min) for each treatment group. Control nests: gray color, linear *R*² = 0.018. Heated nests: black color, linear *R*² = 2.712E−6.

On day 3 of nestling age, the intensity of *Haemoproteus* infection was significantly lower in females from heated nests compared with females from control nests (Table 3; Fig. 3). However, the intensity of *Lankesterella* infection was not significantly affected by the experiment, although we found a trend to be lower in females from heated nests (Table 3). In addition, we found that in control nests, females that incubate for longer periods exhibit higher *Haemoproteus* and *Lankesterella* infection intensities (Tables 4 and 5; Figs. 4 and 5).

**Table 3:** Differences between treatment groups on the intensity of infection by blood parasites of female blue tits at day 3 of nestling age

	Control	Heat	Statistic	*P*
***Haemoproteus***	21.17 ± 8.48	5.9 ± 2.37	*F*_1,39_ = 4.923	**0**.**033**
***Lankesterella***	2.62 ± 1.24	0.6 ± 0.405	*F*_1,39_ = 3.362	0.074

Estimated marginal means ± SE are shown. Statistics correspond to results of GLMs. Significant results are marked in bold.

**Table 4:** Results of a GLM analysing the relationship between abundance of *Haemoproteus* in females (on day 3 of nestling age) and their average incubation session duration (min), treatment and their interaction

	B	Statistic	*P*
**Average incubation session duration (min)**	0	*F*_1,40_ = 2.132	0.144
**Treatment**	(Control nests) = −0.043	*F*_1,40_ = 5.653	**0**.**017**
**Treatment × Average incubation session duration (min)**	(Control nests × Average incubation session duration (min)) = 0.083	*F*_1,40_ = 34.421	**<0**.**001**

Estimated marginal means ± SE are provided. Significant results are marked in bold.

**Table 5:** Results of a GLM analysing the relationship between abundance of *Lankesterella* in females (on day 3 of nestling age) and their average incubation session duration (min), treatment and their interaction

	*B*	Statistic	*P*
**Average incubation session duration (min)**	−0.027	*F*_1,40_ = 2.883	0.090
**Treatment**	(Control nests) =0.499	*F*_1,40_ = 0.357	0.550
**Treatment × Average incubation session duration (min)**	[Control nests × Average incubation session duration (min)] = 0.106	*F*_1,40_ = 25.350	**<0**.**001**

Estimated marginal means ± SE are provided. Significant results are marked in bold.

Considering the data from days 3 and 13 of nestling age, the interaction between treatment and date was not significant for *Haemoproteus* (GLMM, *F*_1,58_ = 0.025, *P* = 0.876; *F*_1,58_ = 0.607, *P* = 0.439; respectively; Tables S6 to S7 and Figure S6). However, in the case of *Lankesterella,* the interaction between experiment and the date had a significant effect (GLMM *F*_1,60_ = 3.633, *P* = 0.018; Tables S8-S9 and Figure S7). Specifically, *Lankesterella* abundance was higher in females from control nests on day 3 (3.313 ± 1.657) than in females from heated nests on day 3 (0.267 ± 0.187) of nestling age.

## Discussion

In this study, we manipulated nest box temperature during the entire incubation period of the Blue Tits. As a result, we increased the temperature by an average of 3.29 °C in heated nests as compared with control nests. This notable increase in temperature could serve as a realistic simulation of a heatwave during the incubation period. As observed in recent years, heatwaves are becoming increasingly frequent, often characterized by rapid and dramatic rises in temperature (Corregidor-Castro et al. 2023). However, it is worth noting that these results are based on data collected over a single year, which may present certain limitations, as environmental conditions can vary annually. For instance, in a study where we manipulated the temperature inside nest boxes during nestling development in 2021 (García-del Río et al. 2025), the average temperatures in control nests (19.48 ± 0.82 °C) were lower than those recorded in the present year (20.65 ± 1.32 °C). Nevertheless, both years were also included in a separate long-term study spanning 21 yrs (Merino et al. 2025), which concluded that they did not differ significantly from the long-term average conditions observed in the study population.

We have found that the behavior of incubating females and their overall incubation effort are highly influenced by the nest microclimate (Amininasab et al. 2016; Arct et al. 2022; Duncan et al. 2024). An experimental increase in nest box temperature resulted in a decrease in the time spent incubating and a decrease in the intensity of infection and the number of blood parasites in Blue Tit females (see Figs. 2–5). In particular, females in heated nests spent less time incubating and had a higher number of incubation sessions than control females. This could be because a higher temperature would keep the eggs warm for longer and prevent them from cooling down as quickly as in the case of control nests during a recess (Ar and Sidis 2002). This allowed females from heated nests to spend less time incubating and to interrupt incubation sessions more often. Other studies also found that increased nest temperatures can cause a reduction in incubation investment (Davis et al. 1984; Bryan and Bryant 1999; Londoño et al. 2008; Álvarez and Barba 2014; Mueller et al. 2019; Duncan et al. 2024). These behavioral changes in response to experimental increases in nest temperature reflect what occurs naturally when ambient temperatures increase during the day and female birds spend less time incubating their eggs (Haftorn and Reinertsen 1990; Amininasab et al. 2016; García-del Río et al. 2026a). Therefore, the temperature is an important predictor of female incubation behavior, which in turn may affect the body condition of incubating females by decreasing the energetic costs of incubation (Bryan and Bryant 1999). In addition, females from heated nests can feed more frequently and for a longer time since they spend more time away from the nest. Nevertheless, we found no effect of the experimental increase in temperature on the body condition of females. We also failed to detect an effect of the experiment on female's body mass measured at day 3 and 13 of nestling age, although females lost weight as the nestling development progresses as expected (Freed 1981). We also found no differences in the number of feedings by males per hour, indicating that males from heated nests did not reduce the number of feedings even when their partners' incubation effort decreased, although it has been shown that males fed their females more frequently in the nest when ambient temperatures were low (Bambini et al. 2019). However, we cannot rule out completely the possibility that males from heated nests bring less food or food of poorer quality per feeding visit.

Although females did not have any benefit in terms of body condition from reducing their parental effort, we found that those who were able to reduce their investment in incubation due to the experimental increase in temperature experienced a less blood parasite infections and infections of lower intensity. That is, a lower number of infected females and females infected by a lower number of different parasites (ie, mixed infections) as well as lower intensity of infection by *Haemoproteus* and *Lankesterella* parasites. Moreover, intensity of infection by *Lankesterella* parasites increases between day 3 and 13 of nestling age in females from the heated group (Figure S7). In addition, we found that in control nests, females with longer incubation periods exhibited higher infection intensities of *Haemoproteus* and *Lankesterella.* This could be due to the fact that females from heated nests might improve resource allocation to immune responses by diminishing investment in parental care, in this case, in incubation, as explained by the existence of a tradeoff between reproductive effort and immunity (Sheldon and Verhulst 1996).

In line with our findings, numerous studies have observed that parasites can affect female parental effort. For example, female Blue Tits infected with blood parasites during incubation laid significantly smaller clutches than noninfected females, that is, they may have reduced their parental investment due to infection by blood parasites (Merilä and Andersson 1999). Furthermore, Red-winged blackbird *Agelaius phoeniceus* and Pied flycatcher *Ficedula hypoleuca* females with experimentally reduced blood parasite loads were able to spend more time on incubation (Schoepf et al. 2022; García-del Río et al. 2026a; respectively), showing that blood parasites affect bird parental effort.

However, most of the experimental effects on female infection disappear by day 13 of nestling age, except for the number of infected females, which remained lower in heated nests and the abundance of *Lankesterella* parasites that increased in females from both groups but especially in those from the heated nests. These results may suggest that females from heated nests experienced a short-term improvement in health. However, as nestling development progressed, infection levels increased, matching those of the control females. Nonetheless, the effect of the experiment on blood parasite infections could also increase future survival or reproductive success. That is, those females with fewer or lower intensities of infections by haemoparasites may have a greater probability of survival to reproduce again in the following seasons (Martínez-de la Puente et al. 2010). Therefore, the absence of any effect on their current reproduction may indicate that females are maximizing their lifetime fitness. However, as this was a 1-yr study, we were unable to confirm this possibility. Furthermore, we found that the two most abundant parasites, *Haemoproteus* and *Lankesterella*, displayed differing dynamics throughout the breeding period. The prevalence of *Haemoproteus* remained unchanged between the two sampling points, although it has been shown in previous studies that it decreases as the reproductive period progresses (Merino et al. 2000). In contrast, *Lankesterella* showed a significant increase by day 13 of nestling age compared with day 3.

The effort that female birds invest in parental care positively influences the growth and health status of nestlings, as seen in Pied flycatchers (Merino et al. 1996; Moreno et al. 1997). However, we did not find differences in hatching success, nestling body mass, or fledging success, as shown in a similar study with Great tits *Parus major* (Álvarez and Barba 2014). This could be because the decrease in time invested in incubation was compensated by the extra heat provided and because control females compensated for the lower temperatures in their nests by investing more time incubating, thus rendering a similar breeding success for females from heated and control nests.

In conclusion, birds possess the ability to adjust their incubation behavior in response to varying climatic conditions, a strategy that may confer advantages for both their immediate health and future reproductive success. By modifying the time spent incubating, birds can better regulate temperature and protect their offspring, ensuring optimal development. However, the flexibility of this behavioral adaptation is not without its constraints. As climate change continues to drive more extreme and unpredictable weather patterns, birds may face conditions that exceed their current capacity for adjustment. In such cases, more substantial shifts in life-history traits, such as altered breeding timing or even changes in reproductive efficiency, might be necessary for their survival and continued reproductive success as reported in our population study (Merino et al. 2025). This raises concerns about the long-term resilience of bird species, as their ability to adapt to increasingly severe environmental changes may be limited.

## Supplementary Material

arag016_Supplementary_Data

## Data Availability

Analyses reported in this article can be reproduced using the data provided by García-del Río et al. (2026b).
