# Supplementary material for: Impact of increased nest temperature on incubation behavior and female health in Eurasian Blue Tit
Source: Behav Ecol. 2026 Feb 21;37(2):arag016. doi: 10.1093/beheco/arag016 (PMC13016975; doi:10.1093/beheco/arag016)
Supplement: arag016_Supplementary_Data [file arag016_supplementary_data.docx]

**Table S1**. Differences between treatment groups in body mass and reproductive success variables. Estimated marginal means ± SE are shown. Statistics correspond to results of GLMs.

|  | **Control** | **Heat** | **Statistic** | ***P*** |
| --- | --- | --- | --- | --- |
| **Hatching success** | 0.887 ± 0.034 | 0.871 ± 0.033 | F_1,42_=0.115 | 0.736 |
|  |  |  |  |  |
| **Fledging success** | 96.58 ± 2.256 | 96.10 ± 2.294 | F_1,42_=0.023 | 0.881 |
|  |  |  |  |  |
| **Female body mass on day 3** | 10.38 ± 0.113 | 10.22 ± 0.118 | F_1,42_=0.928 | 0.341 |
|  |  |  |  |  |
| **Nestling body mass on day 3** | 2.933 ± 0.100 | 3.089 ± 0.100 | F_1,42_=1.450 | 0.235 |

**Table S2.** Model estimates (GLMM) of the variation in female’s body mass in relation to sampling date and experimental manipulation of temperature. Significant results are marked in bold.

|  |  | | | | |
| --- | --- | --- | --- | --- | --- |
|  | | **Coeficient** | **Statistic** | ***P*** |  |
| **Date** | | (Day 3) = 0.255 | F_1,84_=18.483 | **<0.001** |  |
| **Treatment** | | (Control nests) = 0.120 | F_1,84_=0.684 | 0.410 |  |
| **Treatment*Date** | | (Control nests*Day 3) = 0.038 | F_1,84_=0.485 | 0.488 |  |
| **Tarsus length** | | 0.050 | F_1,84_=0.166 | 0.410 |  |

| **Table S3.** Residual effect of variance components of female’s body mass GLMM. | | | | | | | |
| --- | --- | --- | --- | --- | --- | --- | --- |
| **Residual effect** | **Estimate** | **SE** | **Z** | ***P*** | **Confidence Interval at 95%** | |  |
|  |  |  |  |  | Lower | Upper |  |
| **Var(Day 3)** | 0.305 | 0.066 | 4.650 | <0.001 | 0.200 | 0.464 |  |
| **Var(Day 13)** | 0.249 | 0.057 | 4.360 | <0.001 | 0.159 | 0.390 |  |
| Subject specification: NEST | | | | | | | |

**Table S4.** Model estimates (GLMM) of the variation in nestlings body mass in relation to sampling date and experimental manipulation of temperature. Significant results are marked in bold.

|  |  | | | | |
| --- | --- | --- | --- | --- | --- |
|  | | **Coeficient** | **Statistic** | ***P*** |  |
| **Date** | | (Day 3) = -7,047 | F_1,86_= 1605.35 | **<0.001** |  |
| **Experiment** | | (Control nests) = -0.051 | F_1,86_=0.029 | 0.865 |  |
| **Experiment*Date** | | (Control nests*Day 3) = -0.041 | F_1,86_=0.014 | 0.907 |  |

**Table S5.** Residual effect of variance components of nestlings’ body mass GLMM.

| \|  \| \| \| \| \| \| \| \| --- \| --- \| --- \| --- \| --- \| --- \| --- \| \| **Residual effect** \| **Estimate** \| **SE** \| **Z** \| ***P*** \| **Confidence Interval at 95%** \| \| \| Lower \| Upper \| \| **Var(Day 3)** \| 0.724 \| 0.153 \| 4.743 \| <0.001 \| 0.479 \| 1.095 \| \| **Var(Day 13)** \| 0.659 \| 0.146 \| 4.528 \| <0.001 \| 0.427 \| 1.016 \| \| Subject specification: NEST \| \| \| \| \| \| \| |
| --- | --- | --- | --- | --- | --- | --- | --- | --- | --- | --- | --- | --- | --- | --- | --- | --- | --- | --- | --- | --- | --- | --- | --- | --- | --- | --- | --- | --- | --- | --- | --- | --- | --- | --- | --- | --- | --- |

**Table S6**. Model estimates (GLMM) of the variation in *Haemoproteus* infection intensity in relation to sampling date (day 3 versus day 13 of nestling age) and experimental manipulation of temperature.

|  | **Coeficient** | **Statistic** | ***P*** |
| --- | --- | --- | --- |
| **Treatment * Date** | (Control nests*Day 3) = 0.612 | F_1,60_=0.949 | 0.423 |

**Table S7.** Residual effect of variance components of *Haemoproteus* infection intensity GLMM.

| \|  \| \| \| \| \| \| \| \| --- \| --- \| --- \| --- \| --- \| --- \| --- \| \| **Residual effect** \| **Estimate** \| **SE** \| **Z** \| ***P*** \| **Confidence Interval at 95%** \| \| \| Lower \| Upper \| \| **Var(Day 3)** \| 1.049 \| 0.275 \| 3.808 \| <0.001 \| 0.627 \| 1.755 \| \| **Var(Day 13)** \| 0.775 \| 0.204 \| 3.808 \| <0.001 \| 0.463 \| 1.297 \| \| Subject specification: NEST \| \| \| \| \| \| \| |
| --- | --- | --- | --- | --- | --- | --- | --- | --- | --- | --- | --- | --- | --- | --- | --- | --- | --- | --- | --- | --- | --- | --- | --- | --- | --- | --- | --- | --- | --- | --- | --- | --- | --- | --- | --- | --- | --- |

**Table S8.** Model estimates (GLMM) of the variation in *Lankesterella* infection intensity in relation to sampling date and experimental manipulation of temperature. Significant results are marked in bold.

|  | **Coeficient** | **Statistic** | ***P*** |
| --- | --- | --- | --- |
| **Treatment * Date** | (Control nests*Day 3) = 0.322 | F_1,60_=3.633 | **0.018** |

**Table S9.** Residual effect of variance components of *Lankesterella* infection intensity GLMM.

| \|  \| \| \| \| \| \| \| \| --- \| --- \| --- \| --- \| --- \| --- \| --- \| \| **Residual effect** \| **Estimate** \| **SE** \| **Z** \| ***P*** \| **Confidence Interval at 95%** \| \| \| Lower \| Upper \| \| **Var(Day 3)** \| 0.983 \| 0.258 \| 3.808 \| <0.001 \| 0.587 \| 1.644 \| \| **Var(Day 13)** \| 1.084 \| 0.285 \| 3.808 \| <0.001 \| 0.648 \| 1.813 \| \| Subject specification: NEST \| \| \| \| \| \| \| |
| --- | --- | --- | --- | --- | --- | --- | --- | --- | --- | --- | --- | --- | --- | --- | --- | --- | --- | --- | --- | --- | --- | --- | --- | --- | --- | --- | --- | --- | --- | --- | --- | --- | --- | --- | --- | --- | --- |


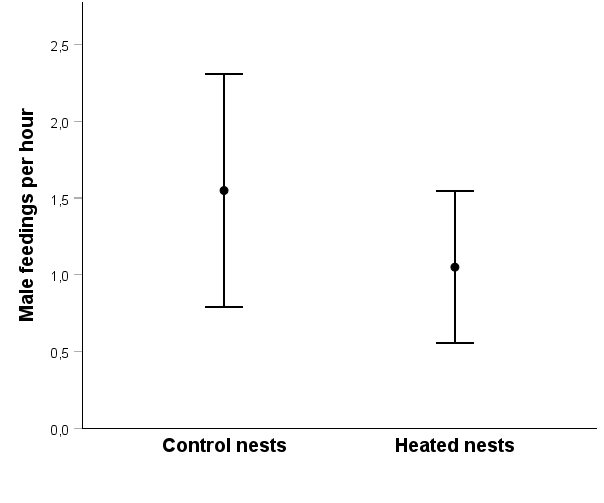


**Figure S1**. Male provisioning per hour to female Blue Tits attending nest boxes assigned to different treatments. Estimated marginal means ± intervals of confidence at 95% are shown.


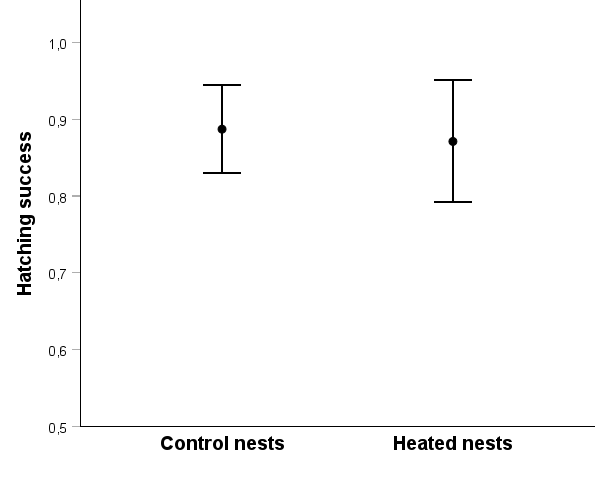


**Figure S2**. Hatching success of clutches from nest boxes assigned to different treatments. Estimated marginal means ± intervals of confidence at 95% are shown.


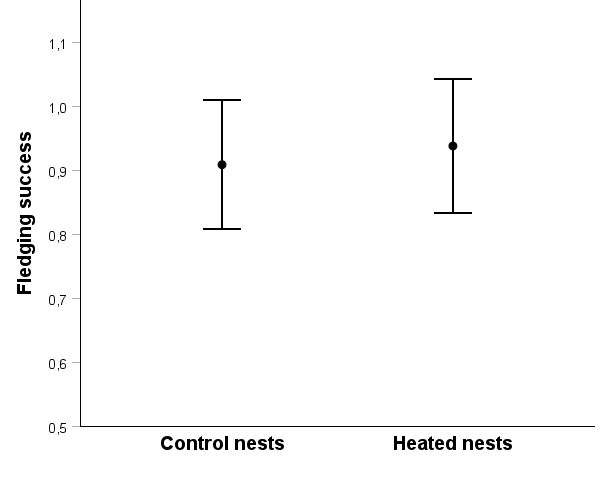


**Figure S3**. Fledging success from nest boxes assigned to different treatments. Estimated marginal means ± intervals of confidence at 95% are shown.


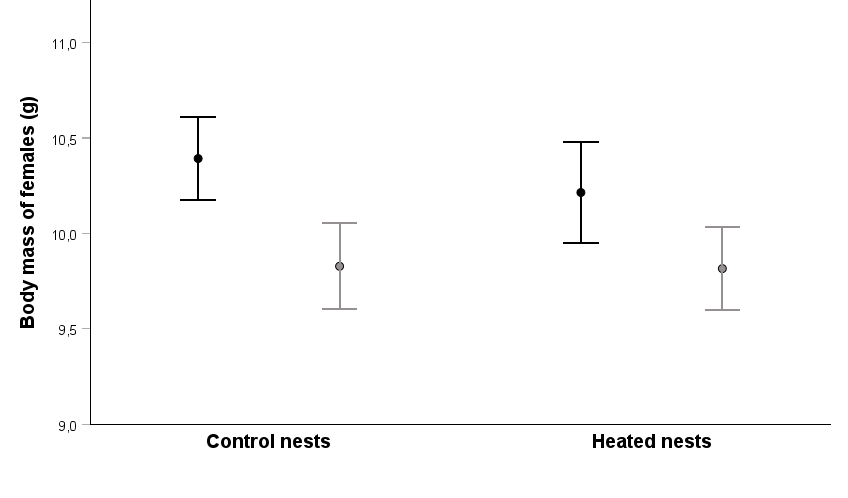


**Figure S4**. Body mass of females attending nest boxes assigned to different treatments, in relation to sampling date (age of nestlings). Estimated marginal means ± intervals of confidence at 95% are shown. Day 3: black colour. Day 13: grey colour.


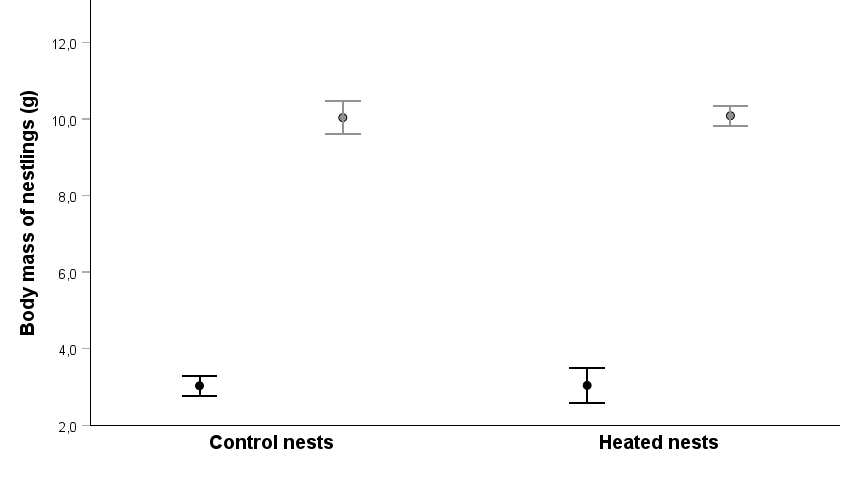


**Figure S5**. Body mass of nestlings from nest boxes assigned to different treatments, in relation to sampling date (age of nestlings). Estimated marginal means ± intervals of confidence at 95% are shown. Day 3: black colour. Day 13: grey colour.


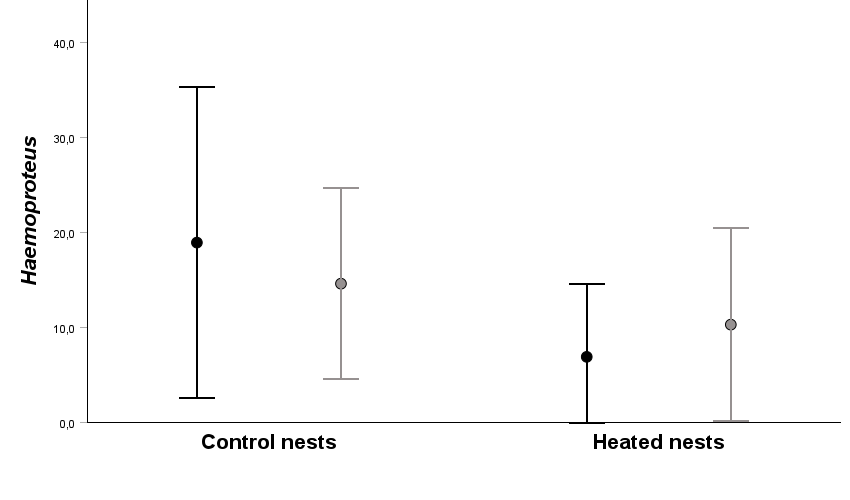


**Figure S6**. intensity of *Haemoproteus* infection in females from nest boxes assigned to different treatments, in relation to sampling date (age of nestlings). Estimated marginal means ± intervals of confidence at 95% are shown. Day 3: black colour. Day 13: grey colour.


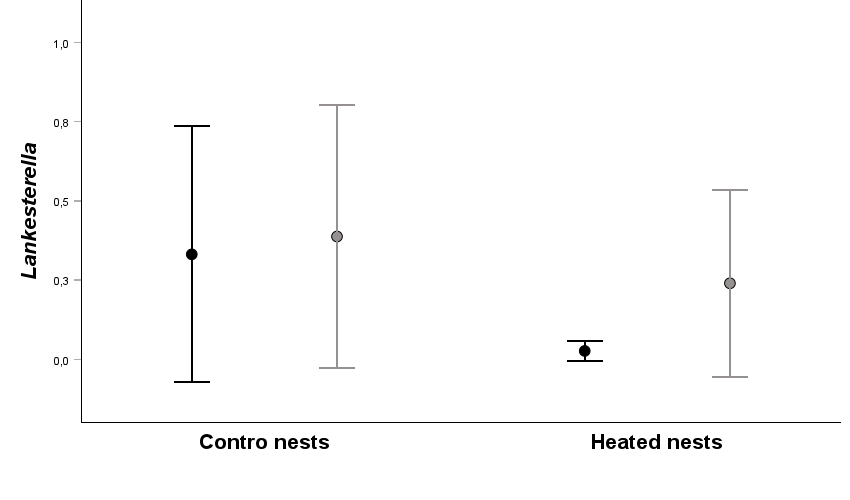


**Figure S7**. Intensity of *Lankesterella* infection in females from nest boxes assigned to different treatments, in relation to sampling date (age of nestlings). Estimated marginal means ± intervals of confidence at 95% are shown. Day 3: black colour. Day 13: grey colour.
